# A prognostic model for cervical cancer based on ferroptosis-related genes

**DOI:** 10.3389/fendo.2022.991178

**Published:** 2022-10-14

**Authors:** Huijun Du, Yumei Tang, Xiaoying Ren, Fan Zhang, Wei Yang, Le Cheng, Yunan Gao

**Affiliations:** ^1^ National Health Commission (NHC) Key Laboratory of Molecular Probes and Targeted Diagnosis and Therapy, Harbin Medical University, Harbin, China; ^2^ Department of Cardiology, The Fourth Affiliated Hospital of Harbin Medical University, Harbin, China; ^3^ School of Basic Medical Sciences, Dali University, Dali, China; ^4^ Shanxi Keda Research Institute, Taiyuan, Shanxi, China; ^5^ Department of Endocrinology, The Fourth Affiliated Hospital of Harbin Medical University, Harbin, China; ^6^ BGI-Yunnan, Kunming, China

**Keywords:** ferroptosis, prognostic model, bioinformatics, differentially-expressed genes, ceRNA

## Abstract

**Background:**

Ferroptosis is widely involved in the occurrence and development of various cancers, but a specific mechanism involving ferroptosis in cervical cancer is still unclear.

**Methods:**

Based on the expressions of ferroptosis-related genes, a prognostic model was constructed using lasso regression, and the overall predictive performance of this model was verified. An in-depth analysis of the prognostic model was then conducted.

**Results:**

The prognostic model showed good predictive performance in both the validation and test sets. Mechanism analysis indicated that differences in the tumor microenvironment were the basis of the predictive ability of the model. Notably, CA9 mRNA was significantly overexpressed in cervical carcinoma, tissues but not in normal cervix tissues. A pair of ceRNAs (CA9/ULBP2) could be involved in the carcinogenesis and development of cervical cancer, and the potential target might be hsa-miR-34a. In addition, predicted miRNAs and drugs for these DEGs were identified.

**Conclusions:**

We constructed a prognostic model with good predictive performance, based on the expression of ferroptosis-related genes. Further research found that the ceRNA pairs of ULBP2/CA9 could regulate cervical cancer through hsa-miR-34a. These results identified the mechanism of ferroptosis in cervical cancer, and might provide novel therapeutics for cervical cancer patients.

## Introduction

Cervical cancer is the fourth most common malignancy diagnosed in women worldwide, and findings have reported that the incidence of cervical cancer is increasing in younger age groups (1, 2). Globally, there are more than 500,000 new cases of cervical cancer annually. This places a heavy burden on female health and economic development. Despite advances in the diagnosis and assessment of cervical cancer, there are no effective therapeutic targets and molecular markers (3).

Tumor microenvironment is a complex dynamic system formed by the interaction between tumor cells and their surrounding cells. It includes immune cells, stromal cells, blood vessels, and the extracellular matrix. It is widely recognized that the tumor microenvironment plays an important role in tumorigenesis and malignant progression (4, 5).

Ferroptosis is a form of regulated cell death that can be inhibited by both iron deficiency and lipophilic antioxidants (6). The increased dependence of cancer cells on iron compared with normal cells results in a satisfactory response to iron therapy, which is why iron can effectively kill cancer cells, while leaving healthy cells intact (7, 8). Ferroptosis has been reported to be extensively involved in multiple malignancies, such as ovarian cancer, kidney cancer, liver cancer, and so on (9–12). In addition, CDC25A was found to inhibit autophagy-dependent ferroptosis in cervical cancer cells (13). Oleanolic acid promotes ACSL4-dependent ferroptosis and inhibits proliferation of cervical cancer cells (14). Together, these studies suggest that ferroptosis is a potential novel diagnostic and therapeutic target for cervical cancer patients.

## Materials and methods

### Data source

Cervical squamous cell carcinoma and endocervical adenocarcinoma (CESC) transcriptome data were downloaded. There were 296 cancer tissue samples, including 283 samples with complete overall survival information, and three control samples from UCSC Xena (https://xenabrowser.net/datapages/). At the same time, clinical data related to files from UCSC Xena (https://xenabrowser.net/datapages/) were also downloaded.

### Identification of differentially-expressed genes

The “DESeq2” package of R studio (https://www.rstudio.com/) was used to identify differentially expressed genes (logFC > 2, P < 0.05) (15). Ferroptosis-related genes were obtained from the FerrDb V1 website (16) (http://www.zhounan.org/ferrdb/legacy/). The online Venn diagram tool (https://bioinformatics.psb.ugent.be/webtools/Venn/) was used to identify common DEGs, including 58 genes.

### Construction of a prognostic model

The “survival”, “glmnet”, “survminer”, and “timeROC” packages of R studio were used to construct a prognostic model of cervical cancer using LASSO regression (17, 18). A new risk score was calculated, and patients were divided into low and high groups based on the optimal truncation values of risk scores. Survival curves, hazard ratio, and receiver operating characteristic (ROC) curve analysis were used for the evaluations of predictive models. Univariate and multivariate analyses were conducted using online websites (http://vip.sangerbox.com/home.html). Kaplan-Meier plotter (https://kmplot.com/analysis/index.php?p=background) was used to analyze associations between the expressions of the DEGs involved in the model and relapse-free survival in patients (the internet site contained 304 cervical cancer samples with complete overall survival information). Univariate COX regression analysis was performed for DEGs and risk scores.

### Enrichment analysis

The “clusterProfiler” package of R studio was used to perform the GO functional enrichment analysis (19).

### Immune infiltration, tumor microenvironment score, and related mechanisms

The “e1071” package of R studio was used for analysis of immune-cell infiltrates, then box plots and heat maps were drawn. To identify the mechanism, GSEA analysis was conducted based on the analysis results. The “estimate” package of R studio was used to score the tumor microenvironment. An online website (https://www.xiantao.love/products) was used to draw scatter plots. Finally, the correlations between DEGs and immune cells were analyzed.

### Expression of proteins

We obtained the expression of proteins from the HPA database of normal and cancer cervical tissues.

### Prediction of miRNA, drugs, and competing endogenous RNAs

The CyTargetLinker application of Cytoscape was used to predict miRNAs and drugs (20). The mRNAs that could form ceRNA pairs with CA9 were predicted using the ENCORI website, and each mRNA was verified by GEPIA website.

## Results

**Figure 1A:**
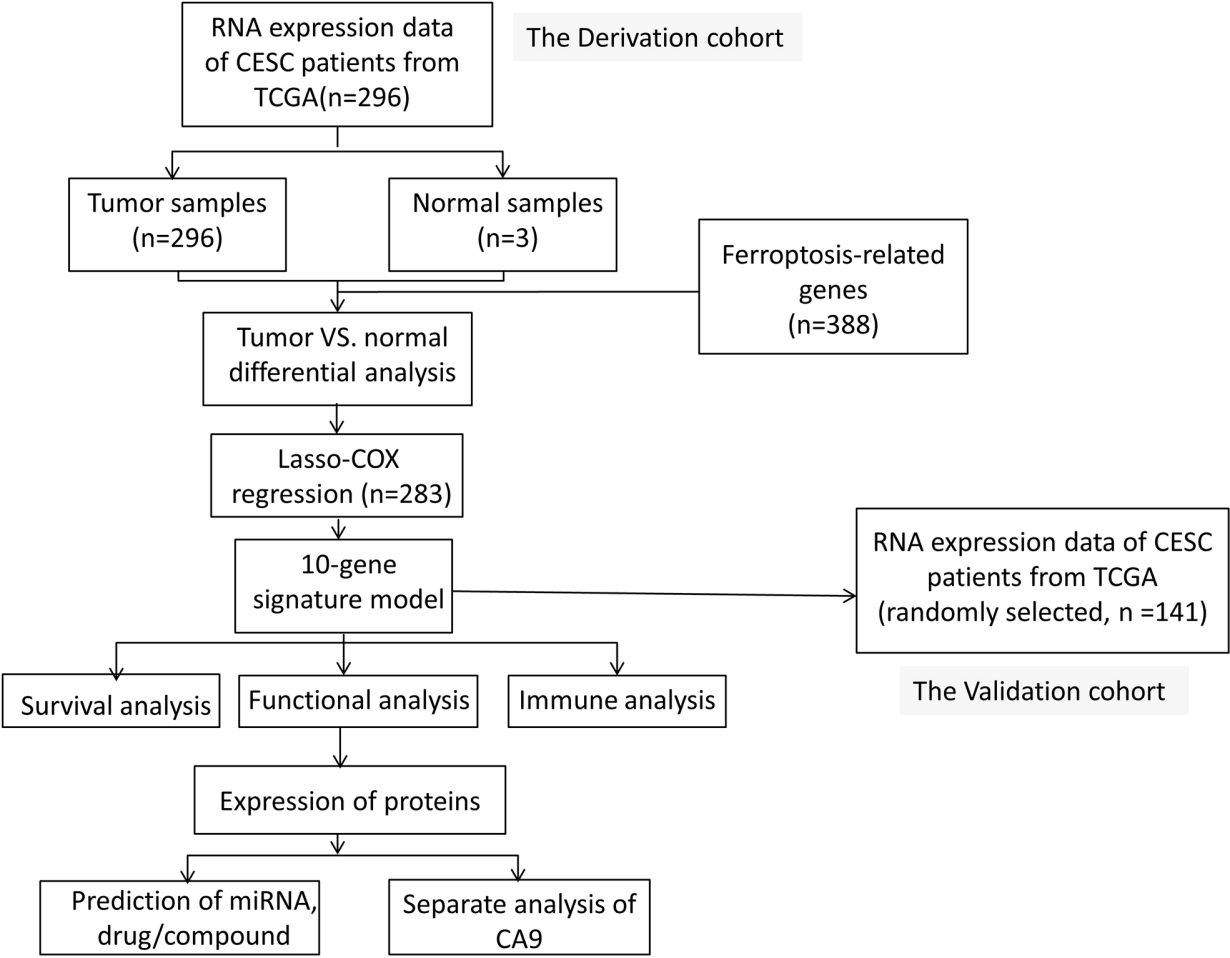
Flowchart depicting the construction and validation of data collection and analysis.

**Figure 1** shows a flowchart depicting the construction and validation of data collection and analysis. The baseline clinical characteristics of the cervical cancer patients in this study are summarized in **Table 1**.

**Table 1:** The distribution of clinical characteristics of cervical cancer patients.

Characteristic	Overall
No.of patients	283
Age (median, range)	46 (20-88)
Stage (%)	
I	154
II	63
III	39
IV	21
Unknown	6
T	
Tis	1
1	134
2	67
3	16
4	10
Unknown	16
N	
0	125
1	54
Unknown	64
M	
0	107
1	10
Unknown	122
Human papillomavirus type, n (%)	
HPV sample	12 (4.2%)
Normal	271 (95.8%)
Survival status	
OS days (median)	715

### Results of DEG identification

**Figure 2:**
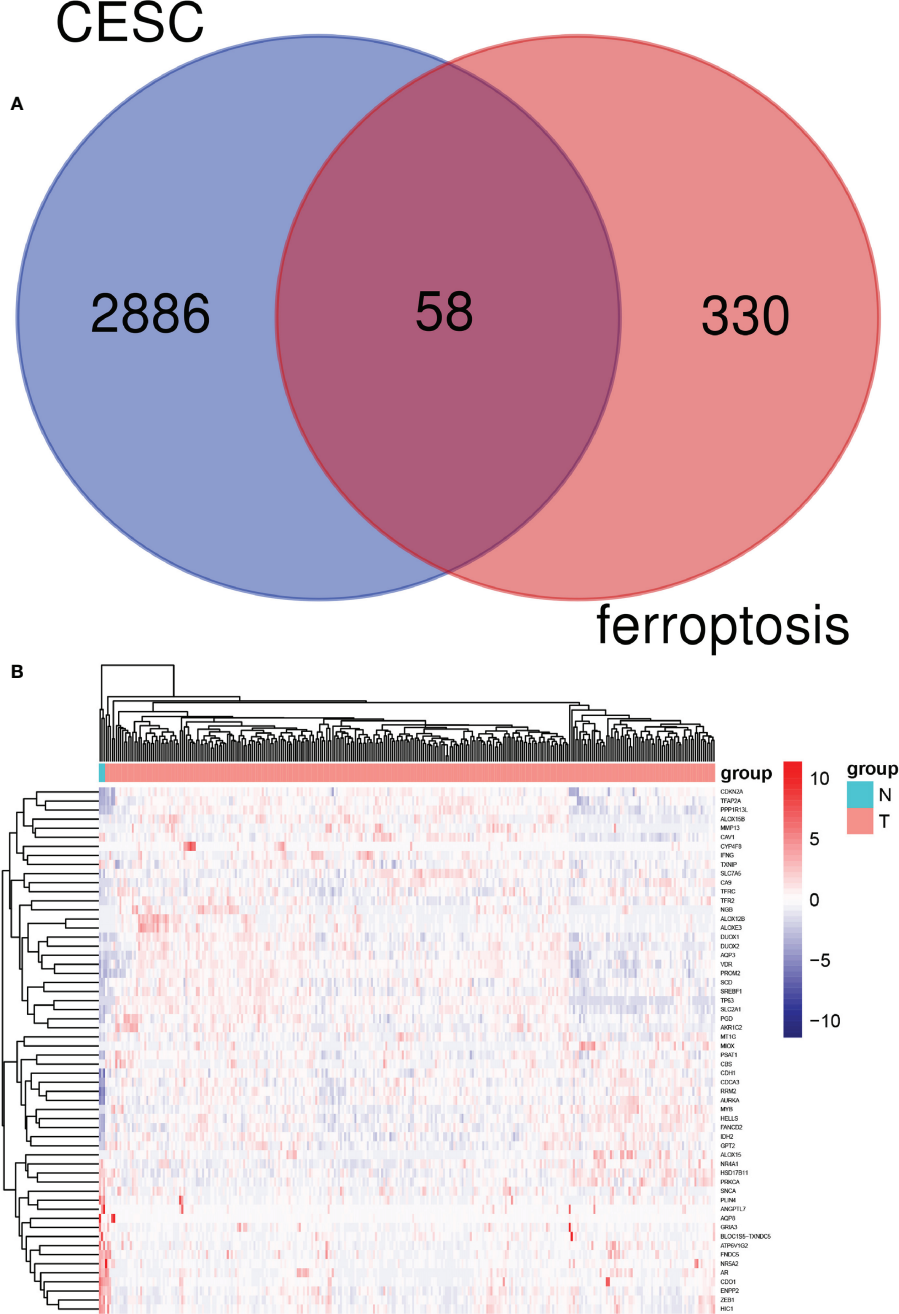
Identification of candidate genes related to ferroptosis in cervical cancer patients. **(A)** Venn diagram illustrating prognostic differentially-expressed genes (DEGs) between cervical cancer and adjacent non-cancer samples. **(B)** Heat map analysis of 58 prognostic DEGs.

The “DESeq2” package of R studio was used to analyze DEGs, with a total of 2,945 DEGs identified in cervical cancer samples. In addition, 388 ferroptosis-related genes were obtained from the FerrDb website. The online Venn diagram tool was used to obtain their common DEGs, including 58 genes, with 39 genes upregulated and 19 downregulated (**Figure 2**).

### A prognostic model based on 58 DEGs

**Figure 3:**
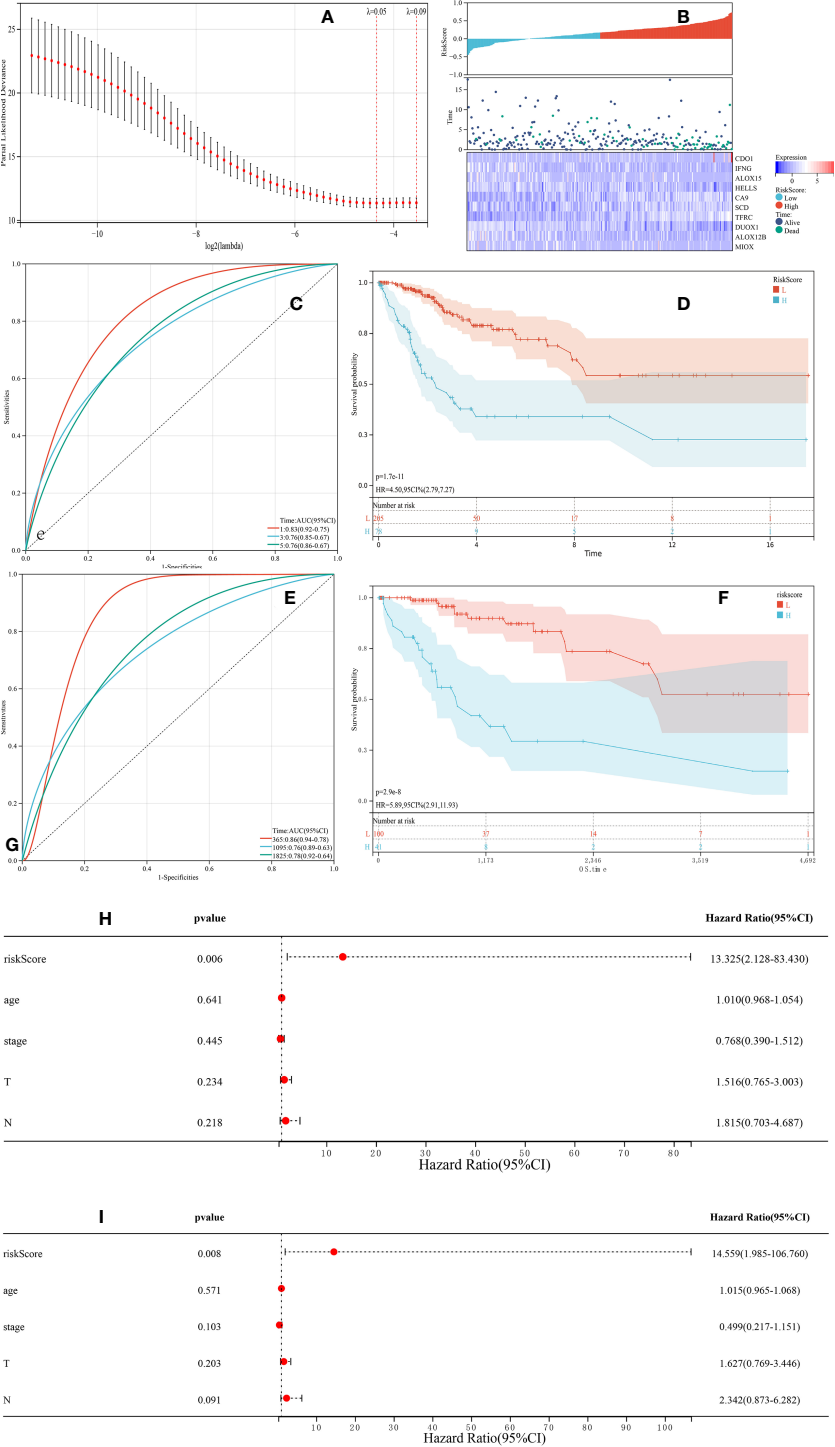
Synthetic analysis of the prognostic value of the ferroptosis-based risk signature. **(A)** The least absolute shrinkage with 10-fold cross-validation, by conducting candidate shrinkage to build the model. **(B)** Distribution of risk scores among endocervical adenocarcinoma samples and 10 gene risk scores and patient survival status. **(C, E)** Time-dependent receiver operating characteristic curves for survival prediction with area under the curve values in both The Cancer Genome Atlas (TCGA) training **(C)** and testing **(E)** cohorts. **(D, F)** Kaplan-Meier curves for the overall survival (OS) of patients in the high risk and low risk group in both the TCGA training **(D)** and testing **(F)** cohorts. **(H, I)** The univariate and multivariate connections of risk scores and other common clinical factors with OS.

**Figure 4:**
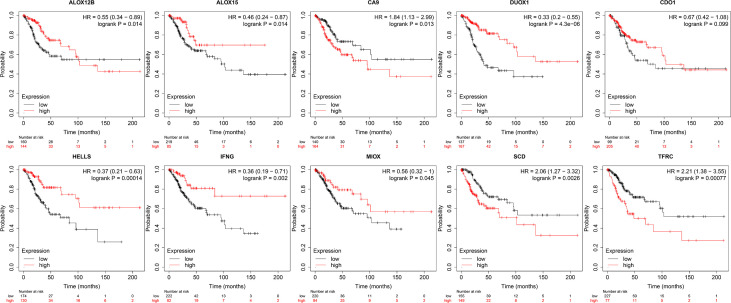
Kaplan-Meier curves of 10 prognostic genes. From left to right: the 1st row shows K-M curves of ALOX12B, ALOX15, CA9, DUOX1, CDO1; the 2nd row displays the K-M curves of HELLS, IFNG, MIOX, SCD, TFRC.

To predict patient outcomes, we performed a randomized LASSO based on 58 ferroptosis-related DEGs, with 10 DEGs identified (including IFNG, ALOX15, HELLS, DUOX1, ALOX12B, MIOX, CDO1, CA9, TFRC, and SCD). The formula for the prognostic risk assessment score was as follows: risk score = (0.0311095071122192*CDO1 expression values - 0.0841228095855146*IFNG expression values - 0.0326830529208614*ALOX15 expression values - 0.100793956921583*HELLS expression values + 0.0259688181408289*CA9 expression values + 0.0197630043617326*SCD expression values + 0.115012577849817*TFRC expression values - 0.07613292120113*DUOX1 expression values - 0.0359912661288295*ALOX12B expression values - 0.0426531549606751*MIOX expression values. Patients were divided into low and high risk groups, followed by prognostic analyses. The results showed that the survival probability of the low risk group was higher than that of the high risk group (P < 0.05), and the area under the curve (AUC) values for OS at 1, 3, and 5 years were 0.83, 0.76, and 0.76, respectively (**Figures 3A–D**). A total of 141 patients were randomly selected to validate the performance of the prognostic model. Similarly, the survival probability of the low risk group was higher than that of the high risk group (P < 0.05), and the AUC values for overall survival at 1, 3, and 5 years were 0.86, 0.76, and 0.78, respectively (**Figures 3E, F**). Univariate (**Figure 3H**) and multivariate (**Figure 3I**) Cox regression analyses showed that the risk score was an independent prognostic factor for all patients with CESC in The Cancer Genome Atlas (TCGA). Further Kaplan-Meier survival analysis revealed that patients with low levels of expression of CA9, SCD, and TFRC mRNA had a better prognosis than those with high expression (**Figure 4**). Together, the results suggested that CA9, TFRC, and SCD were risk factors for cervical cancer (**Figure 3**).

### Enrichment analysis

**Figure 5:**
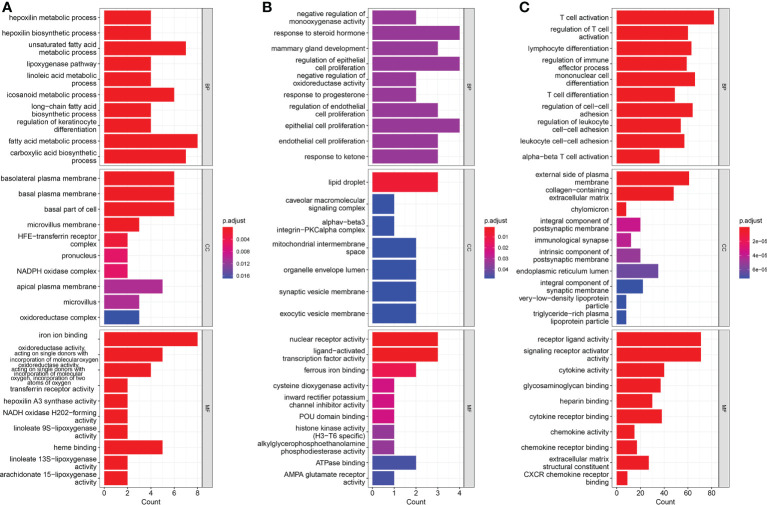
Results of Gene Ontology (GO) pathway enrichment analyses of differentially-expressed genes (DEGs) among **(A)** up-regulated genes and **(B)** down-regulated genes. **(C)** Results of GO pathways enrichment analyses of DEGs among low risk and high risk groups.

To better understand the function of DEGs, Gene Ontology (GO) functional analysis was conducted. A total of 39 up-regulated genes were mainly enriched in GO functions associated with iron ion binding, fatty acid metabolic processes, carboxylic acid biosynthetic processes, and unsaturated fatty acid metabolic processes (**Figure 5A**). A total of 19 down-regulated genes were mainly enriched in GO functions associated with response to steroid hormones, regulation of epithelial cell proliferation, epithelial cell proliferation, lipid droplets, nuclear receptor activity, and the ligand-PKC-alpha complex (**Figure 5B**). Enrichment analysis based on the risk scores showed that the main enrichment pathways were T cell activation, receptor ligand activity, and signaling receptor activator activity (**Figure 5C**).

### Tumor microenvironment and its related mechanism

**Figure 6:**
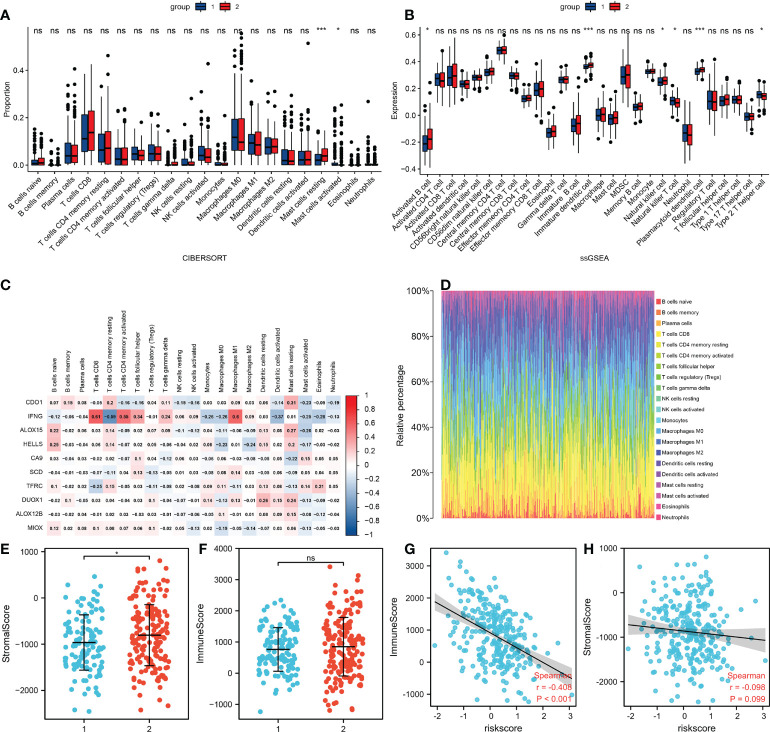
Differences in the tumor microenvironment. **(A)** The infiltration levels of 22 immune cell subtypes in the high and low risk groups. **(B)** Quantitation of distinct immune cell subsets using ssGSEA. **(C)** The correlation between 10 FRGs and immune cells. **(D)** Immune cell proportions for each tumor patient. **(E)** Relationship of stromal scores between the two groups. **(F)** Relationship of immune scores between the two groups. **(G)** Correlations between risk and immune scores. **(H)** Correlations between risk and stromal scores. *p<0.05 ***p<0.001 Ns: no significance.

To further understand the relationship between risk score and immune microenvironment, the “e1071” package was used to analyze immune cell infiltrates (**Figure 6D**). The microenvironment score was obtained using the “Estimate” package, which showed that there was significant difference in stromal score between the two groups, with the same results with immune scores (**Figures 6E, F**). In addition, there was a significant negative correlation between risk score and immune score (P <0.001), but no correlation between risk score and stromal (**Figures 6G, H**). We also found significant cell differences in the circulating levels of resting mast cells and activated mast cells, activated B cells, immature dendritic cells, natural killer cells, natural killer T cells, plasmacytoid dendritic cells, and type 2 T helper cell (**Figures 6A, B**). We then analyzed the correlations between 10 ferroptosis-related genes and immune cells (**Figure 6C**). In conclusion, the results suggested a significant difference in immune microenvironment between the two groups, which explained the different prognoses between the two groups of patients.

### Protein expression levels (original images from the HPA dataset)

**Figure 7:**
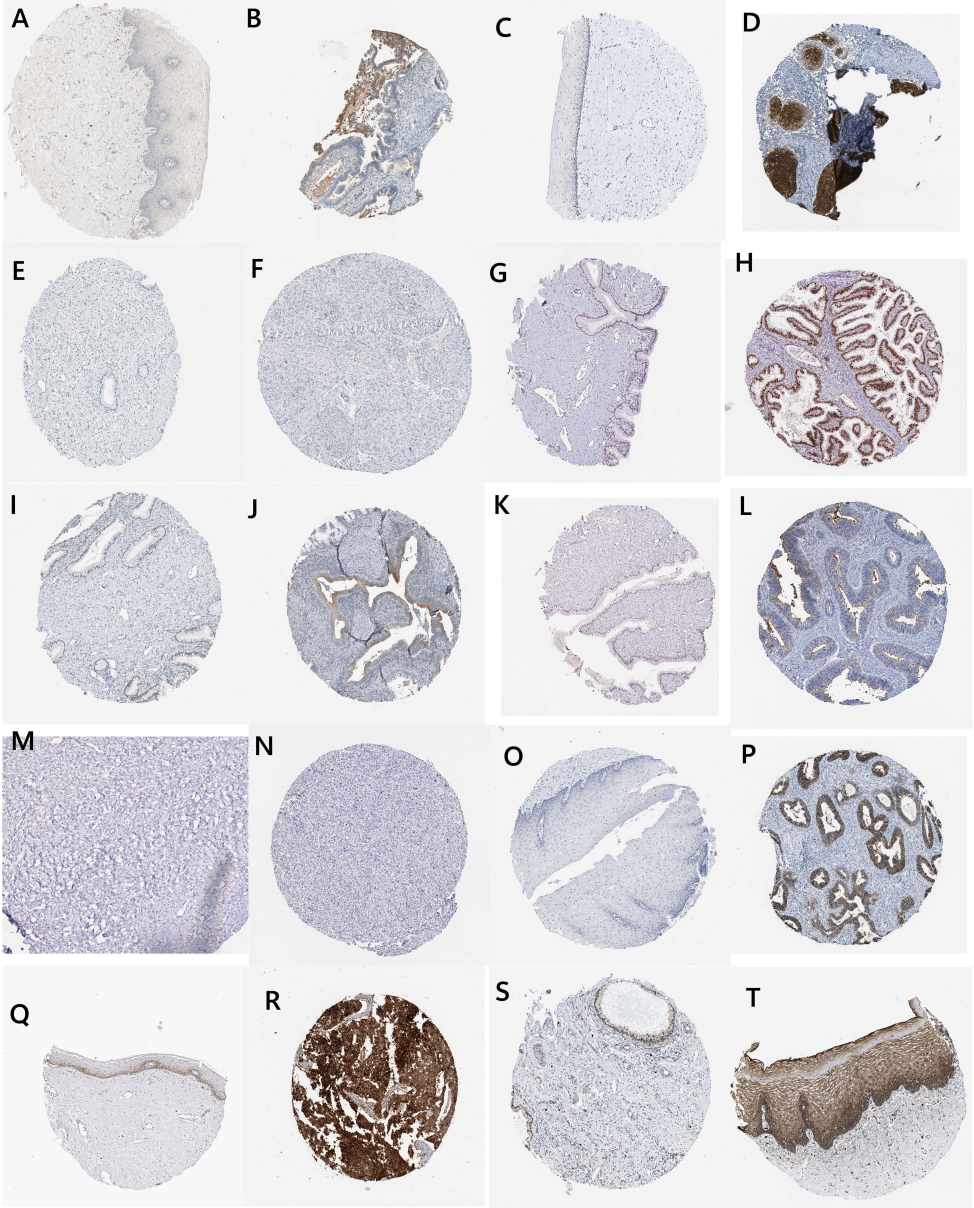
Histological and immunohistochemical images (IFNG: **A**, normal; **B**, tumor ALOX15; **C**, normal; **D**, tumor ALOX12B; **E**, normal; **F**, tumor HELLS; **G**, normal; **H**, tumor DUOX1; **I**, normal, **J**, tumor MIOX; **K**, normal; **L**, tumor CDO1; **M**, normal; **N**, tumor CA9; **O**, normal; **P**, tumor TFRC; **Q**, normal; **R**, tumor SCD; and **S**, normal; **T**, tumor).

We obtained the expression levels of secreted proteins in normal and cancer cervical tissues from the HPA database (**Figure 7**). Among them, ALOX12B and CDO1 were not expressed in both normal cervical and cervical cancer tissues. SCD and TFRC were expressed in both normal cervical and cervical cancer tissues, while CA9 was expressed in only cervical cancer tissues. The results suggested that CA9 played a crucial role in the occurrence and development of cervical cancer.

### Predicting potential drugs and miRNAs

**Figure 8:**
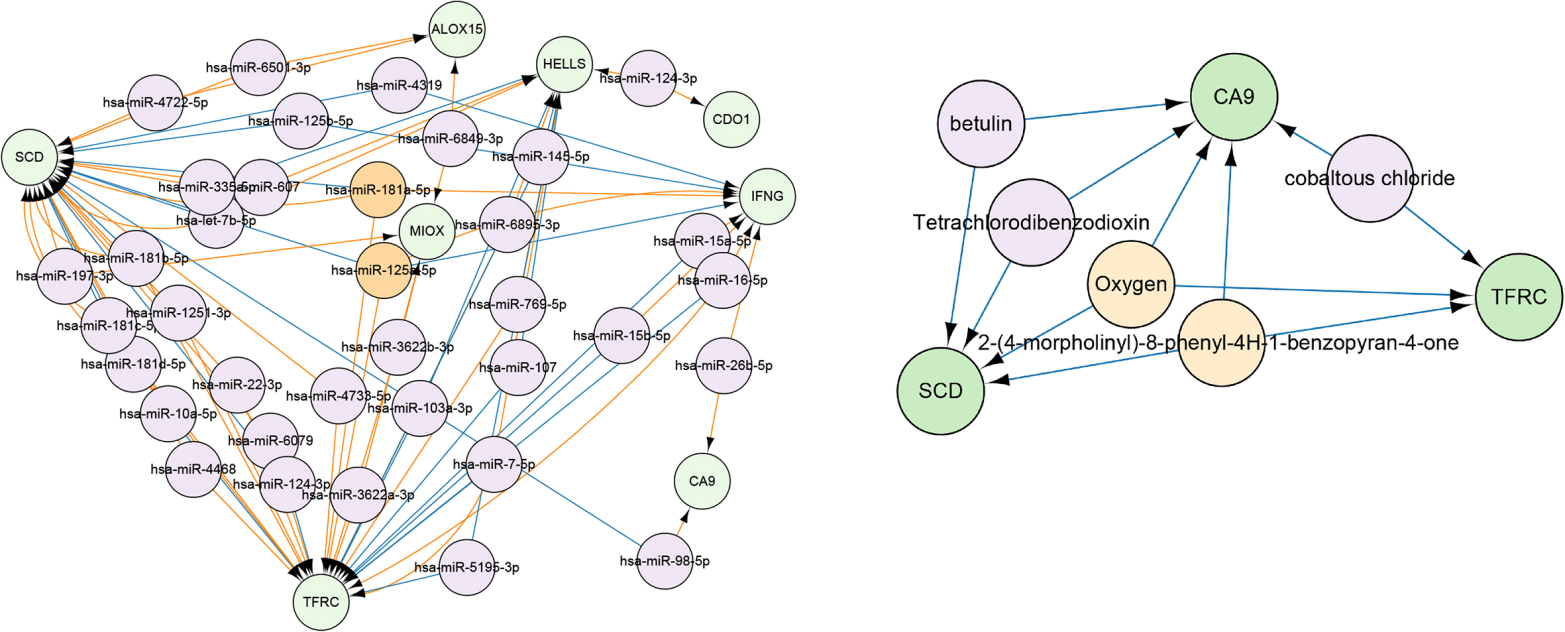
Green modules indicate gene names, and orange modules indicate miRNA and drugs/compounds that could regulate three or more genes simultaneously. The purple module shows miRNA and drugs/compounds that could modulate only two genes.

The CyTargetLinker application of Cytoscape was used to predict miRNAs that targeted DEGs, and to visualize miRNAs that could regulate two or more genes simultaneously (**Figure 8**). Among them, has-miR-125a-5p and has-miR-181a-5p could simultaneously regulate three genes, including SCD, TFRC, and IFNG. Furthermore, we conducted drug predictions for risk factors, which showed that 2-(4-morpholinyl)-8-phenyl-4H-1-benzopyran-4-one and oxygen could act on these three genes (**Figure 8**).

### Comprehensive analysis of CA9

**Figure 9:**
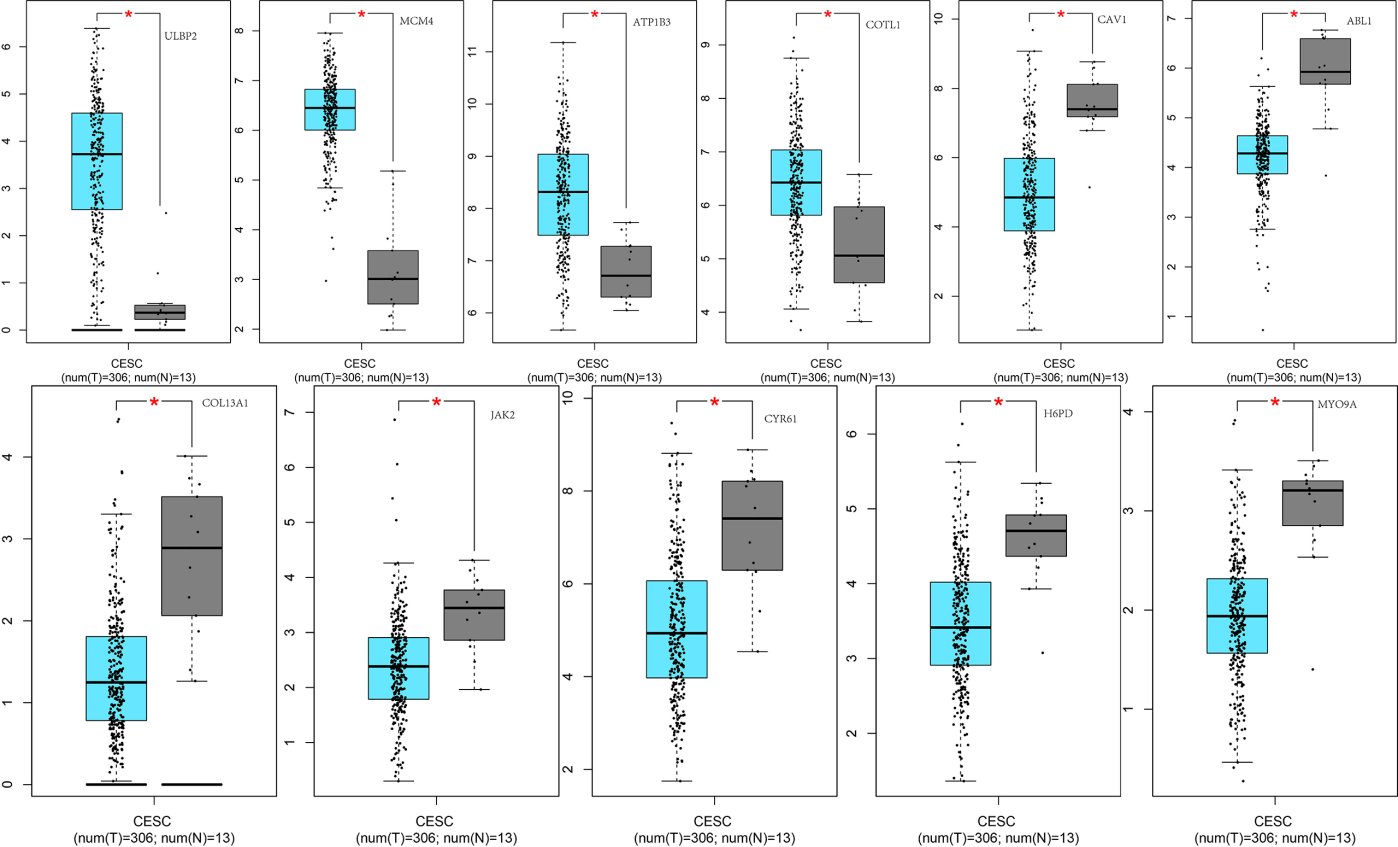
Differential expression of ceRNAs between the two groups of samples. From left to right: the 1st row shows differential expression of ULBP2, MCM4, ATP1B3, COTL1, CAV1, ABL1 between the two groups of samples; the 2nd row displays differential expression of COL13A1, JAK2, CYR61, H6PD, MYO9A, between the two groups of samples. *p<0.05.

**Figure 10:**
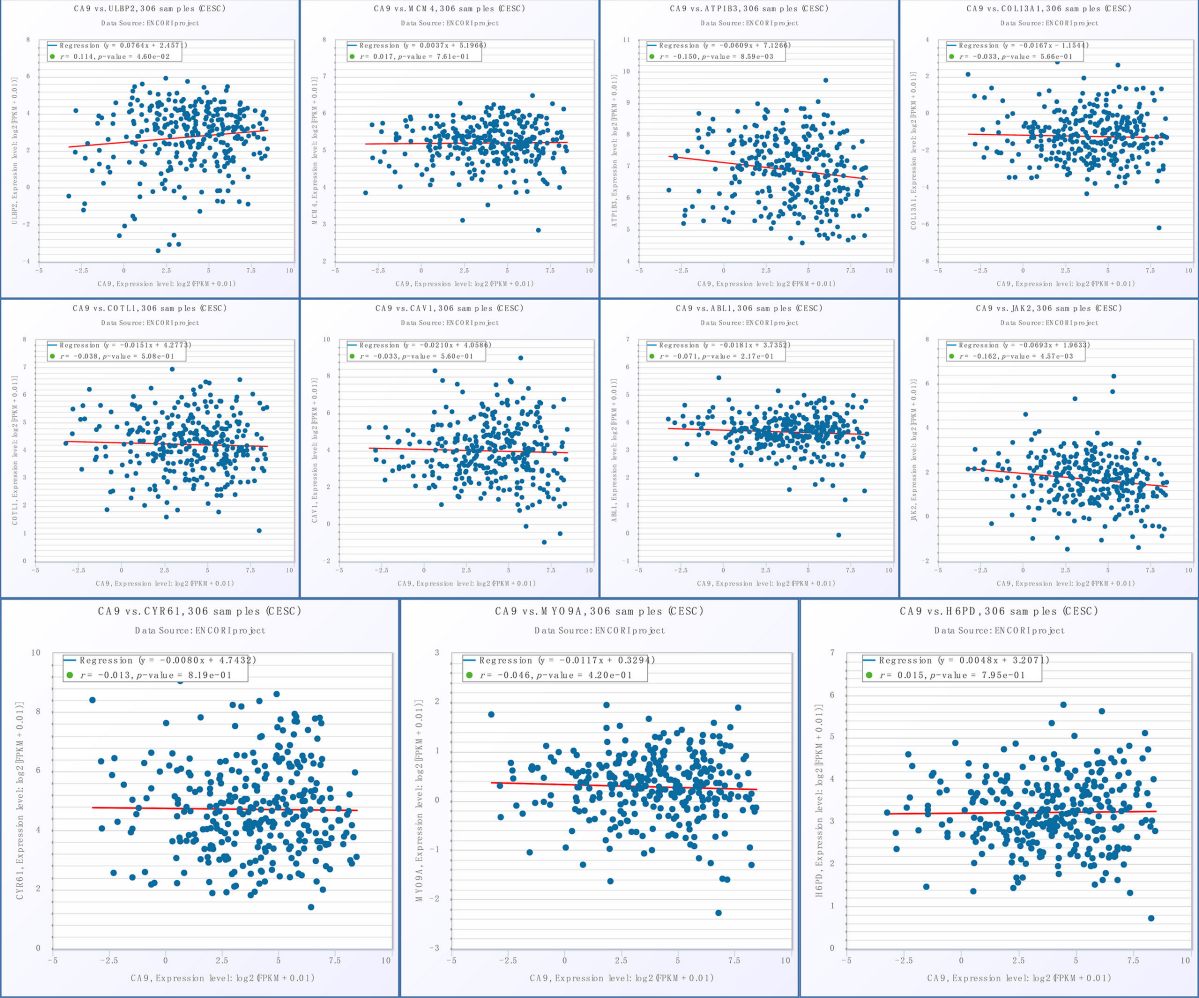
Association of CA9 and ceRNAs. From left to right: the 1st row shows the correlation between CA9 and ULBP2, MCM4, ATP1B3, COL13A1; the 2nd row displays the correlation between CA9 and COTL1, CAV1, ABL1, JAK2; the 3rd row shows the correlation between CA9 and CYR61, MYO9A, H6PD.

Given the important role of CA9, we performed individual analyses for CA9. The mRNAs that could form ceRNA pairs with CA9 were predicted using the ENCORI website, and the expression levels of candidate mRNAs in CESC were verified by GEPIA. The results showed that ABL1, ATP1B3, CAV1, COL13A1, COTL1, CYR61, H6PD, JAK2, MCM4, MYO9A, and ULBP2 were differentially expressed between cervical cancer and normal tissues (**Figure 9**). We then analyzed the associations of CA9 with the expression of these genes, and found that CA9 was positively correlated with ULBP2 (**Figure 10**). CA9 and ULBP2 acted as ceRNAs in CESC by regulating 12 candidate miRNAs, including hsa-miR-1197, hsa-miR-1286, hsa-miR-182-5p, hsa-miR-34a-5p, hsa-miR-34c-5p, hsa-miR-449a, hsa-miR-449b-5p, hsa-miR-4436a, hsa-miR-5000-3p, hsa-miR-556-5p, hsa-miR-1271-5p, and hsa-miR-96-5p. In addition, we suggest that hsa-miR-34a was especially important for CA9. Previous studies confirmed that CA9 and hsa-miR-34a interacted with each other to regulate breast cancer stem cells.

## Discussion

Ferroptosis has been observed in many human malignancies, such as breast cancer, colon cancer, ovarian cancer, liver cancer, glioma, osteosarcoma, and renal cell carcinoma.

The initiation and progression of cancer are inextricably linked to ferroptosis, based on the following. 1) Erastin, a classic ferroptosis activator, was discovered because it triggered cancer cell death. 2) Activation of the Ras-Raf-MEK-ERK pathway was essential for Erastin-induced cell death. 3) Iron was crucial for cancer cell proliferation. 4) The PI3K pathway was one of the most frequently altered signaling pathways in cancer, and cancers with a PI3K-AKT-mTOR pathway mutation were also one of the most difficult cancers to treat. In addition, it was reported that the combination of PI3K/Akt/mTOR pathway inhibitors and ferroptosis-inducing drugs could significantly eliminate tumors (21, 22).

The World Health Organization reported that cervical cancer comprised 12% of all worldwide cancers, and that it was the most common gynecological malignancy in the world. Nearly 84% of cervical cancers occur in the low and middle income countries, placing a heavy burden on women’s health and economies. The main risk factors for cervical cancer include human papillomavirus (HPV) infections, sexual partners, smoking, and long-term use of oral contraceptives. To date, cytology and HPV testing are the most common methods for cervical cancer screening in clinical practice. This cancer screening has greatly improved the detection of early cervical cancer. However, there are no specific biomarkers for early diagnoses and to predict the prognoses of cervical cancer patients. However, microarray and bioinformatics analysis have been widely used to screen for potential diagnostic and prognostic biomarkers, making great contributions to the study of disease genes and molecular mechanisms.

In this study, we constructed a prognostic model using a random lasso method based on ferroptosis-related DEGs, and verified the predictive performance of the model. We also showed that the risk score was an independent risk factor for CESC. Furthermore, we analyzed the model in depth. It was found that the tumor microenvironment was significantly different between high and low risk groups. In addition, the risk score was negatively correlated with immune score, which meant that a lower risk score was correlated with more active immune activity. It was also found that the immune cell types differed between two groups such as those of mast cells, activated B cells, immature dendritic cells, natural killer cells, natural killer T cells, and plasmacytoid dendritic cells.

Mast and dendritic cells were especially important. Significantly increased numbers of mast cells have been found at the sites of many human and murine tumors, including malignancies of the breast, pancreas, lung, and stomach. Studies have found that active substances released by activated and degranulated mast cells in tumor tissues could stimulate tumor growth, metastatic potential, and angiogenesis. The tumor microenvironment also plays an important role in regulating the recruitment of mast cells to tumor tissues, as well as mast cell maturation and activation. More importantly, when comparing benign lesions to malignant lesions, the number and activation of mast cells in cervical epithelium increase, indicating that mast cells play an important role in promoting the gradual malignant transformation of precancerous lesions (23). Dendritic cells (DCs) are the most important antigen-presenting cells. They play a central role in the anti-tumor immune response. After interacting with antigens, immature DCs differentiate into mature DCs that initiate antigen-specific T-cell responses to cancer. DC differentiation and maturation is therefore a key link in antigen-specific anti-tumor immunity. Other studies have found that the number of CD1a+DCs was significantly downregulated in some invasive cervical cancers (24). *In vitro*, the supernatant of SiHa cells (cervical cancer cell line, HPV16+) significantly downregulated DC differentiation (25). Therefore, based on the above study, we suggest that DC differentiation was inhibited in the development or progression of some cervical cancers. Our study was also consistent with this result, showing that immature dendritic and plasmacytoid dendritic cells were significantly downregulated in the poor prognosis group.

Functional enrichment analysis of the DEGs of the two groups revealed that they were involved in signaling pathways and the immune response. In conclusion, the differences in the tumor microenvironment between the two groups contributed to the different prognoses. In addition, we predicted that there might be some miRNAs related to 10 DEGs, and identified miRNAs that may have regulated multiple genes, among them, has-miR-125a-5p and has-miR-181a-5p could simultaneously regulate SCD, TFRC and IFNG.

The miR-125a-5p functions as a tumor suppressor, and its expression is often down-regulated in tumors. It was found that upregulation of miR-125a inhibited hepatocytes proliferation by the STAT3/p-STAT3/JUN/BCL2 axis (26). It also could inhibit breast cancer growth by inhibiting proliferation, to induce autophagy (27). More importantly, exosomal miR-125a-5p expression levels in patients with cervical cancer were significantly lower than those in healthy controls. In addition, miR-125a-5p could inhibit the progression of cervical cancer by down-regulating the activity of GALNT7 and the EGFR/PI3K/AKT signaling pathway. However, miR-181a-5p is more likely to promote tumor progression. The miR-181a-5p can relieve the downstream inhibition of CARM1, thus inactivating the type 1 interferon response in the LUAD and LUSC (28). It also facilitates tumor progression through NDRG2-induced activation of the PTEN/AKT signaling pathway of breast cancer (29) and osteosarcoma (30). More importantly, CCAT1 can promote cervical cancer cell proliferation and invasion by regulating the miR-181a-5p/MMP14 axis (31). The miR-181a-5p can also increase cell proliferation and invasion and inhibit the apoptosis of cervical cancer cells by regulating INPP5A (32). In conclusion, miR-125a-5p and miR-181a-5p have been reported to be involved in the development of cervical cancer, whose mechanism is not yet completely clarified. We used bioinformatics tools to identify a potential mechanism, which may be related to SCD, TFRC and IFNG. However, more studies are necessary to confirm this possibility.

CA9, TFRC and SCD are all risk factors for cervical cancer, so we predicted candidate drugs for these genes, among which 2-(4-morpholinyl)-8-phenyl-4H-1-benzopyran-4-one and oxygen could regulate all three of these risk factors.

The 2-(4-morpholinyl)-8-phenyl-4H-1-benzopyran-4-one, also known as LY294002, is a cell permeable inhibitor of PI3K. Research has found that it can treat gliomas (33), head and neck cancer (34), leukemia (35), breast cancer (36), ovarian cancer (37), and bladder cancer (38). Oxygen is a colorless, odorless, and tasteless gas, and studies have confirmed that hypoxia promotes tumor invasiveness and metastasis (39, 40). Here we found that it also had the potential to treat cervical cancer, in a mechanism involving CA9, TFRC, and SCD. Among them, CA9 is the most widely known hypoxia-inducible factor (HIIF) -1α target gene during hypoxia, which also provides a theoretical basis for oxygen treatment of cervical cancer. However, further experiments are needed to verify this possibility. CA9 is involved in HIF-1-target-gene expression, which can provide the theoretical basis for the use of oxygen as a cervical cancer treatment, but further experiments are required to examine this possibility.

More importantly, we collected proteins from HPA and found that CA9 was highly expressed in cervical cancer tissues, but not expressed in normal cervical tissues. These results indicated that ferroptosis in cervical cancer cells was closely related to CA9, so CA9 could be a potential target for treatment of cervical cancer patients.

Based on the results, we further analyzed CA9, hoping to clarify the mechanism in the involvement of CA9 of ferroptosis in cervical cancer. Based on the ENCORI website, ULBP2 was identified as a ceRNA that might sponge hsa-miR-34a to regulate CA9 and affect CESC prognosis.

The reduced expression of ULBP2 in cervical cancer suppresses the killing effect of Vδ2T cells on cancer cells, and enhances the proliferation, migration, and invasion of cervical cancer cells. In addition, ULBP2 has been shown to be associated with invasive liver tumorigenesis (41), and soluble ULBP2 derived from pancreatic cancer cells can reduce the cytotoxicity of NK cells. Cisplatin can suppress the androgen receptor (AR) expression by increasing miR-34a-5p to suppress AR expression, and the inhibited AR may function through upregulation of ULBP2 protein, thereby enhancing NK cell function and preventing hepatocellular carcinoma progression (42). The platinum-based drugs are also the first-line chemotherapy for cervical cancer, especially cisplatin, which has shown good efficacy in the treatment of cervical cancer patients. Studies have shown that miR-34a directly targeted the 3’-untranslated region of ULBP2 mRNA, and that levels of miR-34a inversely correlated with expression of ULBP2 surface molecules (43). Previous studies have reported that CA9 was directly targeted by miR−34a. In addition, CA9 SNP rs1048638 increases the risk of cervical cancer, which might be due to an allele at CA9 rs1048638, which impairs miR-34a binding to the CA9 3′-UTR and desensitizes CA9 mRNA to miR-34a-dependent RNA degradation, in turn increasing CA9 expression (44). The hsa-miR-34a also regulates both CA9/JAGGED1 and CA9/NOTCH3 ceRNA networks by acting as a miRNA sponge (44, 45).

Most of the 10 DEGs were shown to be involved in ferroptosis in cancer. Combined with results from a previous analysis, we hypothesize that they might be involved in the regulation of ferroptosis in cervical cancer. Targeting ALOX15 and blocking of subsequent reactive oxygen species (ROS) accumulation can inhibit ferroptosis in gastric cancer cells (46, 47). HELLS can interact with WDR76 to up-regulate lipid metabolism, thereby inhibiting ROS accumulation and reducing intracellular iron accumulation, and inhibiting ferroptosis (48). ALOX12B can produce AA/AdA-PE-OOHs peroxidation by PUFA ester peroxidation, which could drive ferroptosis (49). MIOX promotes ferroptosis in HCC cells by regulating the levels of Fe2+ and GSH (50). CDO1 can regulate reduced glutathione and ROS levels of cancer cells to regulate ferroptosis (51). Reducing free iron by targeting TFRC is generally considered a therapeutic strategy for various cancers (52). SCD1 can protect cancer cells from ferroptosis by increasing MUFA synthesis (53). It was found that IFNγ could cooperate with AA to induce tumor cell ferroptosis (54, 55).

A previous research group established a 4-gene signature related to ferroptosis of cervical cancer patients (TFRC, ACACA, SQLE and PHKG2) (56). We also constructed a ten-gene risk fraction model (IFNG, ALOX15, HELLS, DUOX1, ALOX12B, MIOX, CDO1, CA9, TFRC, and SCD) using a similar method. Among them, TFRC gene was our common part, and then we carried out a more detailed and comprehensive analysis. Most importantly, we conducted an in-depth analysis of the ten genes in the model, as well as the related miRNAs and drugs that might have therapeutic potential for cervical cancer. In addition, this is consistent with previous finding that CA9 increased the risk of cervical cancer, we found that CA9 played a crucial role in cervical cancer progression. Subsequently, we wanted to know how CA9 works in cervical cancer, then we found that CA9/ULBP2 ceRNA network may be a key mechanism in the pathogenesis of cervical cancer. hsa-miR-34a might act as miRNA sponge. However, the limitation of our study is that it has not been confirmed by the experimental results, but this is what our research group will do next, hoping to truly enrich the pathogenesis of CA9 of cervical cancer and apply the theory into practice. In conclusion, we constructed a prognostic prediction model associated with ferroptosis, validated the predictive power of this risk model, and then further identified its mechanism, which may be due to the immune microenvironment. In addition, separate analysis of CA9 found that the ceRNA networks (CA9/ULBP2) might regulate the occurrence and development of cervical cancer, but additional experimental results are needed. However, it provided a theoretical basis for treating cervical cancer patients by targeting ferroptosis.

## Conclusion

In this study, we used bioinformatics methods to construct a prognostic model. Further analysis of the model showed that it provided good predictive performance and was closely related to the tumor microenvironment. In addition, CA9 could sponge hsa-miR-34a to promote the progression of cervical cell carcinoma through ULBP2. These results provided potential information regarding the involvement of ferroptosis in cervical cancer, and also provided a theoretical basis for the clinical treatment of cervical cancer patients.

## Data availability statement

Publicly available datasets were analyzed in this study. This data can be found here: https://www.ncbi.nlm.nih.gov/geo/.

## Author contributions

HD and YT prepared the manuscript and the figure. XR, FZ, WY contributed to manuscript conception and design. LC and YG critically reviewed the manuscript.

## Funding

This work was supported by Major Science and Technology Projects of Yunnan Province [Digitalization, development and application of biotic resource, 202002AA100007], and [Study on the construction of comprehensive surveillance and early warning system of acute infectious diseases in Yunnan Province, 202102AA100019]; and Yunnan High-level Talent Training Support Program, special project for industrial technology leading talents [2021, Yunnan Development and Reform Commission]; and Scientific and technological innovation talents project of the Fourth Affiliated Hospital of Harbin Medical University [HYDSYCXRC202122].

## Conflict of interest

The authors declare that the research was conducted in the absence of any commercial or financial relationships that could be construed as a potential conflict of interest.

## Publisher’s note

All claims expressed in this article are solely those of the authors and do not necessarily represent those of their affiliated organizations, or those of the publisher, the editors and the reviewers. Any product that may be evaluated in this article, or claim that may be made by its manufacturer, is not guaranteed or endorsed by the publisher.
